# Bovine Ocular Squamous Cell Carcinoma—A Descriptive Epidemiological Survey in the Azores, Portugal

**DOI:** 10.3390/vetsci13040371

**Published:** 2026-04-11

**Authors:** Beatriz Bilhastre, Helena Vala, Ana Clara Ribeiro, Sara Faria, Ana Oliveira, Sandra Branco, Carlos Pinto

**Affiliations:** 1 Molecular Biology Laboratory, Egas Moniz Center for Interdisciplinary Research (CiiEM) Egas Moniz University Institute, Quinta da Granja, Monte da Caparica, 2829-511 Caparica, Portugal; acribeiro@egasmoniz.edu.pt (A.C.R.); sfaria@egasmoniz.edu.pt (S.F.); 2 MED Mediterranean Institute for Agriculture, Environment and Development & CHANGE Global Change and Sustainability Institute, Institute for Advanced Studies and Research, Universidade de Évora, Portugal; 3 Centro de Estudos de Recursos Naturais, Ambiente e Sociedade (CERNAS), Escola Superior Agrária de Viseu, Instituto Politécnico de Viseu, Av. Coronel José Maria Vale de Andrade, Campus Politécnico, 3504-510 Viseu, Portugal; hvala@esav.ipv.pt; 4 Centro de Investigação e de Tecnologias Agro-Ambientais e Biológicas (CITAB &Inov4Agro), Universidade de Trás-os-Montes e Alto Douro, Quinta de Prados, Apartado 1013, 5001-801 Vila Real, Portugal; 5 Departamento de Biologia Animal, Faculdade de Ciências, Universidade de Lisboa, 1749-016 Lisboa, Portugal; 6 CIISA—Centre for Interdisciplinary Research in Animal Health, Faculty of Veterinary Medicine, University of Lisbon, 1300-477 Lisbon, Portugal; amoliveira@fmv.ulisboa.pt; 7 Associate Laboratory for Animal and Veterinary Sciences (AL4AnimalS), 1300-477 Lisbon, Portugal; 8 MED Mediterranean Institute for Agriculture, Environment and Development & CHANGE Global Change and Sustainability Institute, Department of Veterinary Medicine, School of Sciences and Technology, Laboratory of Pathological Anatomy of the EU Veterinary Hospital, University of Évora, Pólo da Mitra, 7002-554 Évora, Portugal; smbb@uevora.pt; 9 Faculty of Agricultural and Environmental Sciences, University of the Azores, Biotechnology Centre of Azores, 9700-042 Angra do Heroísmo, Portugal; carlos.a.pinto@uac.pt

**Keywords:** bovine ocular squamous cell carcinoma, cattle, Azores, epidemiology, risk factors, ultraviolet radiation (UV), pasture-based management, Holstein Friesian, animal health

## Abstract

Bovine ocular squamous cell carcinoma (BOSCC) is the most common ocular tumour in cattle, with a multifactorial aetiology involving ultraviolet (UV) radiation, genetic factors, pigmentation, and management practices. A detailed epidemiological characterisation of BOSCC in the Azores, Portugal, is provided, with particular emphasis on its spatial distribution and potential risk determinants. Data were obtained through an epidemiological questionnaire completed by field veterinarians between August 2023 and March 2025. A total of 85 BOSCC cases were recorded across 62 farms—45 on Terceira Island and 17 on São Miguel Island. All affected animals were adult Holstein Friesian dairy cows, managed under extensive pasture-based systems. The nictitating membrane was the most frequently affected structure (69.5%), and multiple lesions occurred in 20% of the cases. Farms located at 200–400 m of altitude presented the highest number of cases. Continuous exposure to UV under pasture-based management represents the main environmental risk factor. Although periocular pigmentation may provide partial protection, other environmental and genetic factors can also contribute to tumour development. Artificial insemination is considered a promising preventive tool, enabling genetic selection for protective traits such as periocular pigmentation. This research provides the first regional epidemiological characterization of BOSCC in the Azores, highlighting the interplay among environmental, genetic, and management-related factors in disease occurrence.

## Simple Summary

Bovine ocular squamous cell carcinoma is the most common eye tumour in cattle and can be caused by a combination of sunlight exposure, genetic traits, skin colour around the eyes, and farm management practices. This study is the first to investigate the occurrence of this disease in the Azores, Portugal, and to identify possible risk factors. Information was collected through a questionnaire answered by field veterinarians between 2023 and 2025, covering 85 cases on 62 farms. Most affected cows were adult Holstein Friesian dairy cows kept on pasture, and the nictitating membrane was the area most often affected. Farms located at medium altitudes reported the highest number of cases. Continuous sunlight exposure appears to be the main environmental risk, while natural pigmentation around the eyes may offer some protection. Using artificial insemination to select for protective traits could help prevent the disease. The present work provides an overview of the distribution and causes of eye tumours in cattle in the Azores and highlights the combined role of environmental conditions, genetics, and farm management in disease occurrence.

## 1. Introduction

Bovine ocular squamous cell carcinoma (BOSCC), commonly referred to as “cancer eye,” is the most frequently diagnosed ocular tumour affecting bovines and has been reported worldwide [1,2,3,4,5]. It arises primarily from epithelial cells and can involve various ocular and periocular structures, including the conjunctival surface, sclerocorneal junction, limbus, nictitating membrane, cornea, and the skin of the eyelids [6,7,8,9]. Although the exact etiology of BOSCC remains unclear, several contributing factors have been proposed, including genetic predisposition [10,11,12], nutritional status [13,14], age [10,15], exposure to ultraviolet (UV) radiation [11,15], lack of periocular pigmentation [10,16], and possible involvement of certain viruses [7,17].

In Portugal, this type of tumour has been reported in the Azores archipelago, namely in São Miguel Island and Terceira Island [18,19]. On these Islands, almost all cattle with BOSCC are not approved for human consumption at slaughterhouses [4,19]. This practice reflects official meat inspection criteria under European Union legislation, whereby carcases or parts thereof that present significant pathological alterations at post-mortem inspection, such as extensive tumours or other conditions that may render the meat unsafe or unsuitable for human consumption, are declared unfit for the food chain by the official veterinarian under European Regulation (EU) 2019/627 and as implemented by the Portuguese legislation [20,21]. Furthermore, it was the second-most common tumour found, after urinary bladder tumours, accounting for 21% of carcass rejections [19,22]. Currently, BOSCC is the predominant neoplasm found in Azorean cattle.

While BOSCC has been widely documented in cattle populations across Brazil [1,23], North America [15,24], Australia, and parts of Europe [1], epidemiological studies for the Azores archipelago are very scarce [18], where cattle farming plays a central economic and cultural role.

This study aimed to characterize the epidemiological profile of BOSCC in cattle on the two most populous islands of the Azores, namely Terceira and São Miguel, and to identify potential farm and animal-level risk factors associated with disease development.

## 2. Materials and Methods

### 2.1. Survey

A questionnaire-based survey was conducted to identify suspected cases of BOSCC. The questionnaire was distributed electronically (PDF format) by email to bovine practitioners of the farm associations of each island, Terceira and São Miguel, UNICOL—Cooperativa Agrícola, C.R.L. and Associação Agrícola, respectively. Participating veterinarians were instructed to complete one questionnaire for each suspected BOSCC case encountered during their routine clinical practice. Case reporting was therefore prospective and based on naturally occurring cases, with no predefined number of questionnaires allocated per veterinarian. The total number of complete questionnaires reflects the number of reported cases. The responses were returned electronically by the participants. The responses were collected from August 2023 to March 2025.

The questionnaire was divided into two main sections. The first part aimed to characterize the farm and included geographic location, altitude above sea level and availability of housing facilities for cattle.

When a BOSCC case was reported, the attending veterinarian completed an epidemiological questionnaire and recorded the farm location at least at the village level. When altitude was not directly specified in the questionnaire, open-access topographic maps and digital elevation data (e.g., Google Earth, Google LLC, Mountain View, CA, USA) were consulted to estimate the approximate elevation of each farm based on the reported village location. Farms were subsequently categorized into three predefined altitude groups: low (100–200 m), medium (200–400 m), and high (>400 m). Because elevation may vary within the same village, altitude classification was based on the best available geographic approximation.

Ultraviolet (UV) radiation index data were not directly collected at the farm level within the scope of this questionnaire-based epidemiological survey. No georeferenced coordinates or date–time metadata were recorded for individual cases; therefore, retrospective linkage with UV radiation datasets was not feasible within the present study design. The clinical record of the farm was consulted to determine the presence of previous cases of suspected BOSCC. The second section of the questionnaire collected information related to the animal affected by suspected BOSCC, including date of birth, sex, breed, production purpose, and reproductive system used (artificial insemination or natural mounting). Case data for this study included the identification of affected eye(s) and the presence of periocular pigmentation. Periocular pigmentation was recorded only for eyelid lesions (upper/lower) as “pigmented” or “non-pigmented”, based on visual inspection by the field veterinarian. Pigmentation was not assessed for the nictitating membrane, limbus or canthi. In a subset of eyelid cases, tumour size precluded reliable classification.

**Figure 1:**
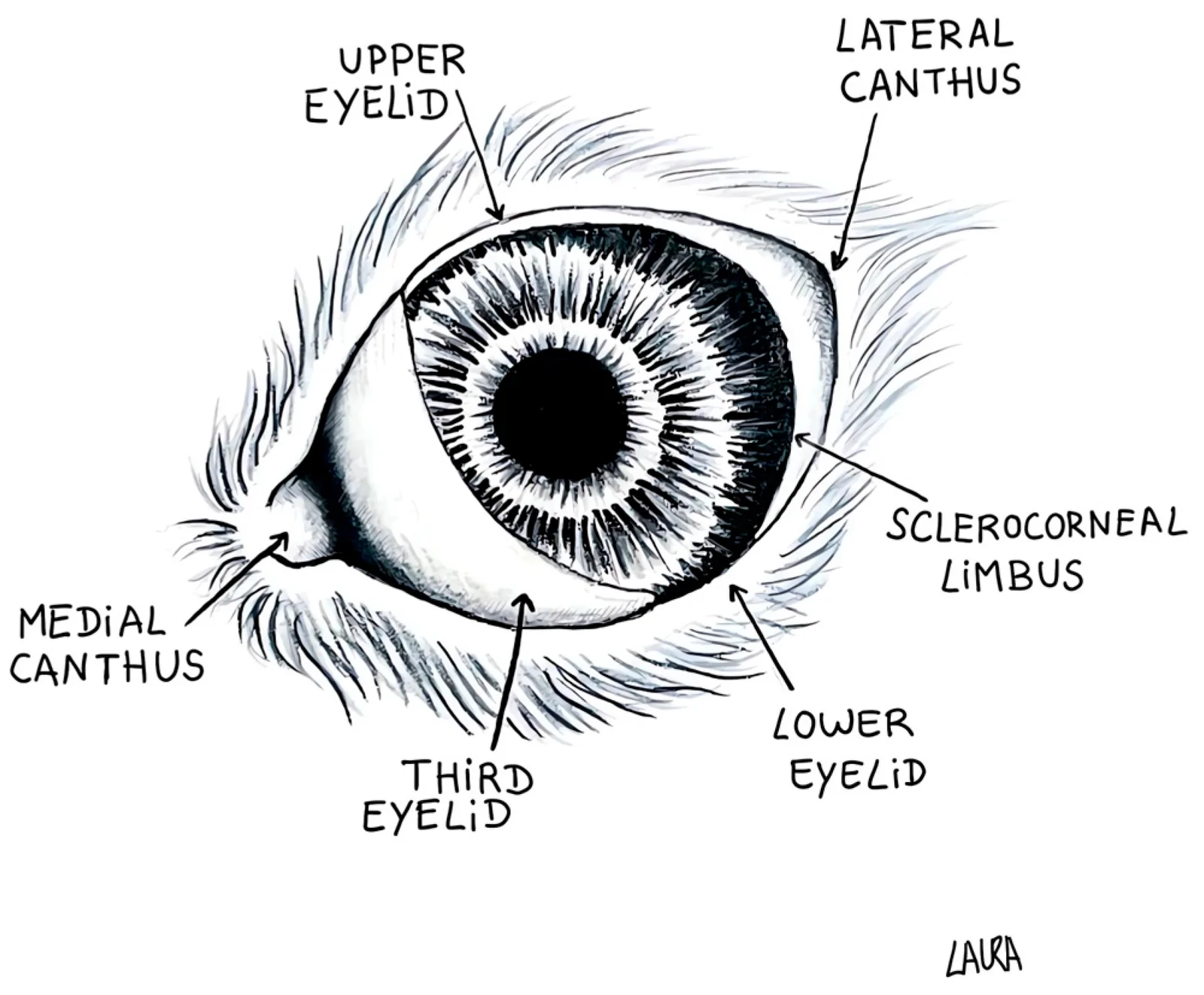
An eye diagram included in the questionnaire for field veterinarians to mark the location of ocular lesions.

For this purpose, a questionnaire included an eye diagram (Figure 1) to facilitate the record of the precise location of the lesion (nictitating membrane, superior or inferior eyelid, medial and lateral canthus, and sclerocorneal limbus).

### 2.2. Case Identification

This study relied on naturally occurring cases in farm settings, with no experimental interventions and no group control, in line with animal welfare regulations. The diagnosis of BOSCC was initially based on clinical assessment performed by field veterinarians at the time of case identification. Veterinarians were instructed to report lesions consistent with classical presentation of BOSCC, including the following: (i) proliferative or ulcerated masses affecting ocular or periocular structures, particularly the nictitate membrane, eyelids, or limbus; (ii) chronic and progressive lesions; (iii) cases occurring predominantly in adult Holstein Friesian dairy cows, a breed known to be predisposed to BOSCC. Inclusion in this epidemiological survey was therefore based on clinical suspicion. Histopathological confirmation was subsequently obtained for most of the cases included in this cohort.

### 2.3. Statistical Methods

Statistical analyses were performed using R software, version 4.3.0 (R Foundation for Statistical Computing, Vienna, Austria). Data normality was assessed using the Shapiro–Wilk test. When the normal distribution was not verified, non-parametric tests were applied. The following variables were included in the statistical analyses: animal-level variable (age), lesion-level variables (location and number), and farm-level variables (altitude and reproduction system).

For comparison of two independent groups, the Wilcoxon rank-sum test (Mann–Whitney U) was used. Categorical variables were analysed using the chi-squared test or Fisher’s exact test, depending on the expected frequencies. Proportions were compared using Z-tests for proportions, and frequency distributions were evaluated through absolute and relative frequencies. A significance level of *p* < 0.05 was adopted for all statistical tests.

## 3. Results

### 3.1. Total Replies

A total of 85 survey responses reporting ocular lesions consistent with BOSCC were obtained, including 64 from Terceira Island and 21 from São Miguel Island. All animals included in this study were adults aged above 1 year old and used for milk production.

### 3.2. Signalment

**Figure 2:**
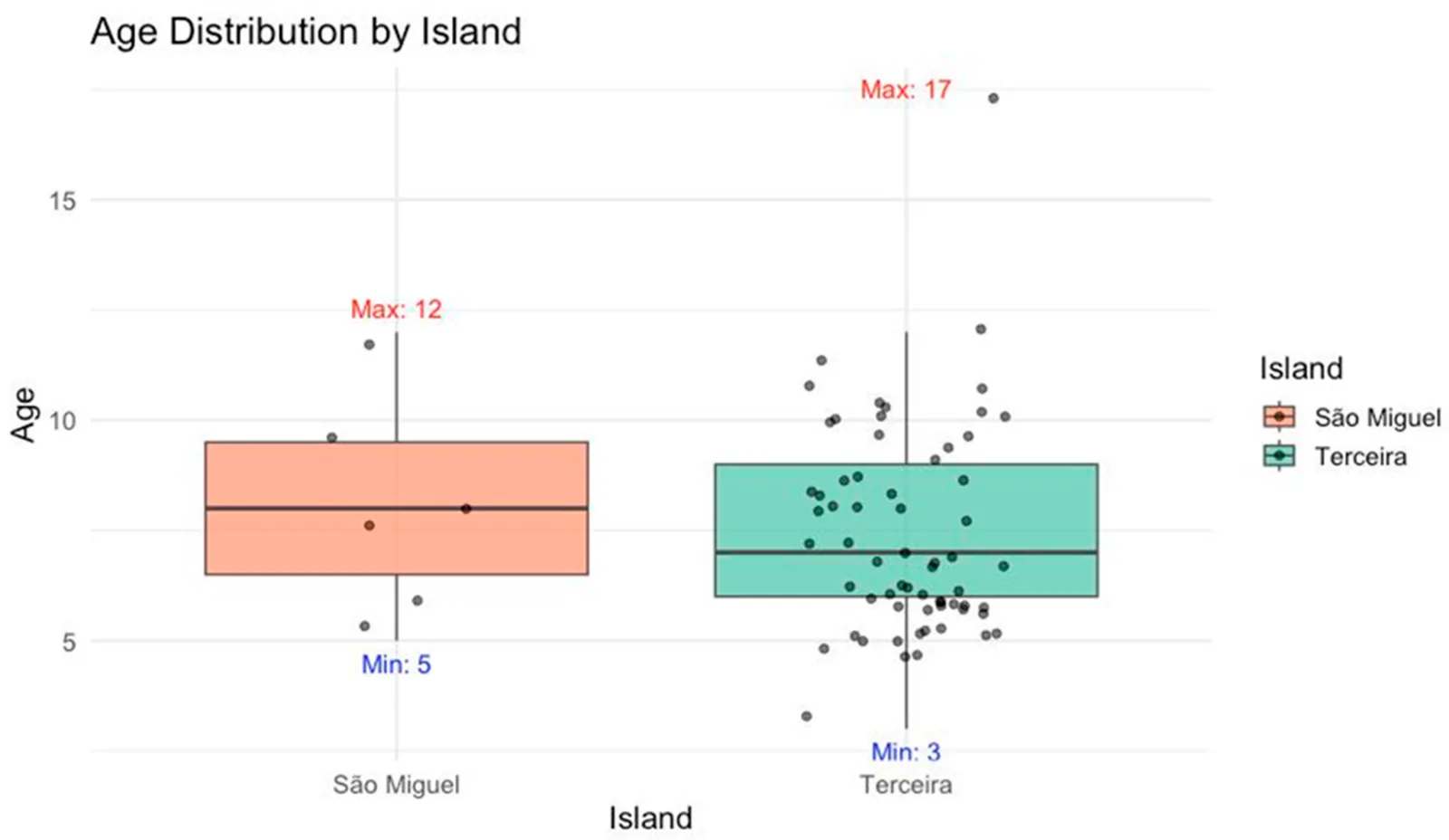
Age distribution of animals affected by suspected BOSCC in both islands.

All the affected animals were Holstein Friesian cows, which are the most common dairy breed in the Azores. One exception was a Montbéliard crossbreed individual located on Terceira Island. The overall median age of the animals was 7.8 years. The age ranged between 5 and 12 years old (median of 8.2 years) for São Miguel Island and 3–12 years old (median of 7.5 years) for Terceira Island. There is no statistical evidence of a significant difference between the ages of the animals from Terceira and São Miguel (*p* = 0.48), and the distribution of the age is shown in Figure 2.

### 3.3. Clinical Results

A total of 85 animals were reported as BOSCC cases. Histopathological confirmation was obtained for 60 of these cases. A total of 105 lesions were recorded across all reported cases, including single and multiple lesions per animal. The frequency of cases with a single lesion was higher (67 cases, 78.8%) than that of cases with multiple lesions (17 cases, 20%). In one case (1.2%), information on the number of lesions was unavailable due to a lack of response to the questionnaire. Within the multilesional subgroup, most of the animals revealed two lesions (14 cases), and rarely three (two cases) or four lesions (one case).

The nictitating membrane was the most affected structure, observed in 73 cases (69.5%), with lesions distributed nearly equally between the left (40%) and right (45.9%) eyes. Other affected structures included sclerocorneal limbus (*n* = 11), inferior eyelid (*n* = 9), medial canthus (*n* = 6), upper eyelid (*n* = 4) and lateral canthus (*n* = 1) and one lesion with unknown location. The distribution of the lesions was similar between both islands (*p* = 1). A total of 13 lesions were located on the eyelids, with eight occurring on non-pigmented skin and five on pigmented skin.

### 3.4. Farm Results

Among the 62 farms included in the study, the frequency of farms reporting a single tumour case was highest, while 18 farms (15 in Terceira and three in São Miguel) reported two tumour cases.

#### 3.4.1. Altitude

Of the 85 suspected BOSCC cases reported through the questionnaire, 38 were from low-altitude areas (44.7%), 46 from medium-altitude areas (54.1%), and only one from a high-altitude area (1.2%) (Table 1).

#### 3.4.2. Previous Reported Cases of BOSCC

Regarding the number of previous BOSCC cases, nine were reported on low-altitude farms and 25 on medium-altitude farms. The severity of the neoplasm cases labelled as ‘‘multiple’’, defined as the presence of more than one ocular lesion, included two occurrences in low-altitude areas and 20 in medium-altitude areas.

#### 3.4.3. Housing Facilities and Management Conditions

Most BOSCC cases were observed in cattle raised under pasture-based conditions. On Terceira Island, all cases (64 animals) occurred in pasture-based dairy systems. Similarly, on São Miguel Island, most cases (14 animals) were in pasture-based animals. In contrast, three cases occurred in stabled animals, and four cows had mixed access to both pasture and stables.

#### 3.4.4. Reproduction System in the Farms

Concerning the breeding method used at the farms, we found that, of the 62 farms surveyed, 32 farms (51.6%) reported using artificial insemination exclusively, while seven farms (11.3%) used natural mating. A total of 22 farms (35.5%) used both methods, although one farm did not provide information regarding the breeding method.

## 4. Discussion

To the author’s knowledge, the present work represents the most comprehensive epidemiological study of BOSCC conducted in the Azores archipelago, and the first report of the potential risk factors associated with this neoplasia in an insular environment. This study was designed as a descriptive epidemiological analysis based on cases as they occurred in clinical practice, reported by veterinary practitioners. Future studies with a control group would allow a more robust data analysis of this disease in the Azores. In the present work, all the animals affected by BOSCC were Holstein Friesian cattle, except for Montbéliard crossbreed individuals in Terceira Island. This is not surprising, since bovine livestock on both islands are largely dominated by this breed with a prevalence above 90% [25]. This result was in accordance with the previous studies’ results [4,26], which reported the highest BOSCC incidence in Holstein Friesian cattle. Similarly, [27] observed the highest BOSCC incidence in Holstein Friesian crossbred animals, followed by Jersey crossbred cows.

In both islands, the neoplasia was mainly diagnosed in adult animals, which is in accordance with previous studies that observed a higher incidence of BOSCC in cattle over 5 years of age. The mean age of the affected animals on both islands to aligns with the general literature; this is in accordance with [28], who reported a mean age of 8 years for cattle with BOSCC. In fact, this type of tumour is uncommon in cattle younger than 5 years and rarely seen in cattle less than 3 years old [4,6,17,27,29].

In our study, the nictitating membrane was the most commonly affected ocular structure, followed by the sclerocorneal limbus and the lower eyelid. These findings are in accordance with [4,6,17], since the authors found that 70% of the animals had lesions at the nictitating membrane and palpebral conjunctiva. These findings contrast with [11], who reported that approximately 75% of BOSCC and precursor lesions were predominantly located on the limbus and cornea, with only 25% observed in the conjunctiva of the eyelids and nictitating membrane. Similarly, in Hereford cattle, [11] identified the most common sites of BOSCC as the lateral and medial corneoscleral junctions. In sum, the ocular structures seem to have different susceptibilities to the development of Squamous Cell Carcinoma (SCC) and play a key role in the pathogenesis of this disease.

Continuous exposure to intense ultraviolet radiation is a recognized risk factor for BOSCC, especially in cattle grazing in fields located in high-altitude areas with sunny climates [29]. Sunlight likely contributes to tumour development by inducing DNA damage, including double-strand breaks and guanine oxidation [30] and by generating reactive oxygen and nitrogen species that cause oxidative and nitrosative stress. These processes lead to lipid peroxidation, protein nitration, and suppression of apoptotic responses, allowing damaged cells to proliferate and promote tumour growth. Markers such as 8-hydroxy-2′-deoxyguanine, malondialdehyde, and nitrotyrosine reflect this cellular damage and have been associated with cancer progression and poorer prognosis [7]. In more aggressive tumours, there is a significant increase in DNA damage markers and, crucially, a suppression or insufficiency of apoptotic responses, allowing damaged cells to continue proliferating and thereby contributing to tumour growth and aggressiveness [30]. The risk is heightened by environmental conditions that increase UV exposure, and while some cellular repair mechanisms exist, they may not fully prevent tumour development under sustained UV stress [31,32,33].

In both islands, the majority of the animals were managed under a pasture-based system. In fact, pasture-based dairy systems are a common practice in the Azores. Such management conditions allow continuous sun exposure, resulting in a relevant cumulative effect of the ultraviolet radiation [34]. As a result, exposure to the carcinogenic effects of sunlight heightened the susceptibility to BOSCC, especially among adult and older cows [4,26]. The occurrence of BOSCC in stabled animals is unusual, as the disease is typically associated with pasture exposure. In our research, three cases were observed in stabled animals. It is possible that these animals had pasture access earlier in life (e.g., during the heifer phase or before parturition), although detailed management histories are not available. This observation highlights the importance of considering lifetime exposure and management practices when interpreting disease distribution.

In this study, carried out on both islands, a higher incidence of cases was observed in farms located at medium altitudes, which is in contrast with other studies that report this condition to be associated with higher altitudes. Considering that both latitude and altitude, together with daily hours of sunlight, contribute to UV radiation exposure, our findings suggest that even at lower altitudes than those typically reported, the combination of latitude-related solar radiation and local climatic and topographic factors may play a significant role in the development of the tumour. However, because the total cattle population at each altitude level was not available in the dataset, altitude should be interpreted as an exploratory variable rather than a definitive risk factor. An overview of previous BOSCC cases across different altitudes suggests that altitude may represent an environmental factor influencing disease risk. Most suspected cases were reported from low- and medium-altitude areas, with very few cases from high-altitude regions. However, altitude was assigned based on village-level location and digital elevation data, and variation in elevation within villages may occur. A total of 85 animals were reported as BOSCC cases, with histopathological confirmation obtained for 60 of these cases [22]. Furthermore, altitude alone does not account for other potentially relevant environmental or management-related factors. Therefore, these findings should be interpreted as descriptive geographic patterns rather than evidence of a direct or causal association between altitude and tumour risk. Future studies incorporating precise georeferenced data and multivariable analyses would help to better clarify the potential role of altitude. Attend to the geographic profile, a volcanic island surrounded by sea, the effect of UV radiation reflection on water can contribute to the increase of incidence in low or medium altitudes.

Regarding the number of previous BOSCC cases, nine were reported on low-altitude farms and 25 on medium-altitude farms. In the present research, the severity of neoplasm cases labelled as ‘’multiple’’, defined as the presence of more than one ocular lesion in the same animal, included two occurrences in low-altitude areas and 20 in medium-altitude areas. Although medium-altitude farms accounted for most of the multiple and clinically severe cases, the presence of severe lesions at lower elevations suggests that altitude alone may not fully explain tumour development in this insular environment.

In this context, Artificial Insemination (AI) may represent a useful tool for the prevention of the disease, allowing genetic selection for periocular pigmentation, as [35] reported that eyelid pigmentation in Hereford cattle has moderate heritability, suggesting that this ocular characteristic can be amplified through selective breeding. This trait gives protection against BOSCC; therefore, its selection can potentially contribute to the reduction in this condition in Frisean Holstein herds. In our study, AI has already been adopted by several farms in both islands.

In cattle, pigmentation occurs in ocular structures, such as the upper eyelid, lower eyelid, nictitant membrane and eyeball [1,36]. The protective role of melanin reduces ultraviolet-induced damage, limiting tumour development [37]. Cattle with unpigmented periocular skin are prone to tumour development, likely because the lack of melanin increases susceptibility to UV-B induced DNA damage [35]. To sustain this idea [38], UV-B radiation, even at low doses, induces significant DNA lesions such as pyrimidine dimers, which are crucial in carcinogenesis. In our study, a few BOSCC lesions were observed in periocular pigmented areas, with five on pigmented eyelids. Because pigmentation was captured solely for eyelid lesions and was missing in some large tumours, these data are not comparable across all anatomical sites and must be interpreted cautiously in a descriptive context. These findings underscore the complexity of BOSCC aetiology and the need to investigate additional risk factors. Future prospective studies where variables like pigmentation around the eyes are systematically documented would allow a more detailed assessment of their role in disease occurrence.

Since pigmentation was assessed only in the upper and lower eyelids, the analysis could not be extended to other ocular structures. Although our comparison between São Miguel and Terceira indicates that periocular pigmentation and the presence or absence of housing facilities are related factors among animals affected by BOSCC, the absence of a control group prevents us from establishing a definitive association or causal relationship. Therefore, while these factors appear correlated with BOSCC cases, further studies including unaffected animals are necessary to confirm any causal link. Ambilateral circumocular pigmentation (ACOP)—pigmentation surrounding the eyes—acts as a natural UV shield, lowering the susceptibility of cattle to BOSCC. As demonstrated by [37], ACOP is a highly heritable trait (h^2^ = 0.79) influenced by multiple genes, including KIT, KITLG, and MITF. Their findings demonstrate that selection for ACOP can effectively reduce the prevalence of BOSCC and support the use of genomic selection, in combination with artificial insemination, to accelerate the spread of protective pigmentation and enhance herd resilience to UV-related ocular diseases.

Although there are no studies correlating unilateral versus bilateral lesion occurrence with prognosis, it is suggested that this clinical data could be incorporated into future studies about prognostic factors in bovines. This highlights the need for further research on lesion multiplicity and prognosis in cattle.

Due to the impact of BOSCC on animal health and productivity, preventive approaches in production animals should primarily focus on genetic selection for periocular pigmentation, a trait with high heritability that may reduce susceptibility to ocular tumours. While regular clinical examinations can support early detection in companion animals, in livestock production systems, the priority should be genetic improvement to prevent the occurrence of the disease and avoid additional surgical costs for producers.

A limitation of the present study is the absence of farm-level ultraviolet radiation index (UVI) data. Although altitude was used as an environmental proxy, it remains an imperfect surrogate of solar UV exposure. While UV irradiance generally increases with elevation, this gradient is influenced by multiple atmospheric and environmental factors, including solar elevation angle, aerosols, surface albedo and cloud cover. The so-called ‘’Background Altitude Effect’’ has been estimated to increase UV radiation by approximately 3–7% per 1000 m of elevation [18]. In the specific context of São Miguel and Terceira islands, where elevations rarely exceed 1000 m, this theoretical increment may fall within the natural variability caused by cloud cover. Publicly available datasets such as TEMIS/KNMI clear-sky UVI products, Copernicus ERA5 reanalysis data and IPMA regional UVI records could enable retrospective exposure modelling in future studies. However, because the present dataset lacks precise farm geolocation and date–time stamps for individual clinical observations, linking UVI to case occurrence would risk exposure misclassification. Integration of these high-resolution geo-temporal data is therefore proposed as an avenue for future research.

A key limitation of this survey is the absence of a control group, which prevents comparison of traits such as periocular pigmentation in non-affected animals and limits the ability to identify risk factors. Studies with limited geographic scope should interpret findings cautiously in terms of generalizability [39]. Consequently, the findings may not be directly applicable to other regions or production systems but may be valuable and informative for future studies conducted in different locations.

## 5. Conclusions

This questionnaire-based survey summarizes the distribution and clinical presentation of BOSCC cases reported in dairy cattle on São Miguel and Terceira. Reports involved adult Holstein Friesian cows mainly kept in pasture-based systems; the nictitant membrane was the most affected site, and single lesions predominated.

Accordingly, no causal inference or risk estimation was attempted. The study provides updated regional epidemiological information and highlights the need for more robust datasets to enable proper risk assessment in future analyses.

Although periocular pigmentation was confirmed as a protective trait, its incomplete effect on this population suggests additional contributing mechanisms, including viral or genetic susceptibility, that warrant further investigation. Importantly, the widespread adoption of artificial insemination offers a practical tool for the dissemination of protective traits such as pigmentation, potentially reducing the long-term burden of BOSCC in dairy herds.

## Figures and Tables

**Table 1:** Distribution of cases by altitude (number of cases), previous cases, and severity of BOSCC.

Altitude	Number of Cases	Previous Cases	Severity (“Multiple”)
Low	38	9	2
Medium	46	25	20
High	1	0	0

## Data Availability

The original contributions presented in this study are included in the article. Further inquiries can be directed to the corresponding author.

## Abbreviations

The following abbreviations are used in this manuscript:

BOSCC	Bovine Ocular Squamous Cell Carcinoma
UV	Ultraviolet Radiation
AI	Artificial Insemination
ACOP	Ambilateral Circumocular Pigmentation
SCC	Squamous Cell Carcinoma
